# Feasibility evaluation of the induced membrane technique with structural autologous strip bone graft management of phalangeal and metacarpal segmental defects using radiography

**DOI:** 10.1186/s12891-023-06519-2

**Published:** 2023-05-25

**Authors:** Jie Fang, Rongjian Shi, Weiya Qi, Dawei Zheng, Hui Zhu

**Affiliations:** Department of Hand Surgery, Clinical Anatomy Laboratory, Xuzhou Renci Hospital, Xuzhou, Jiangsu, 221004 Jiangsu People’s Republic of China

**Keywords:** Induced membrane, Bone graft, Hand, Bone defect

## Abstract

**Purpose:**

The purpose of this study was to explore the feasibility and evaluate the clinical outcomes of treatment for phalangeal and metacarpal segmental defects with the induced membrane technique and autologous structural bone grafting.

**Methods:**

Sixteen patients who sustained phalangeal or metacarpal bone segmental defects were treated by the induced membrane technique and autologous structural bone grafting from June 2020 to June 2021 at our center.

**Results:**

The average follow-up was 24 weeks (range, 12–40 weeks). Radiography demonstrated union of all bone grafts after an average of 8.6 weeks (range, 8–12 weeks). All incisions at donor and recipient sites demonstrated primary heal without infection complications. The mean visual analog scale score of the donor site was 1.8 (range, 0–5), with a good score in 13 cases and a fair score in 3. The mean total active motion of the fingers was 179.9°.

**Conclusions:**

The feasibility of the induced membrane technique and structural treatment with a cylindrical bone graft for segmental bone defects of the metacarpal or phalanx is demonstrated by follow-up radiography results. The bone graft provided much more stability and structural support in the bone defects, and the bone healing time and bone union rate were ideal.

## Introduction

The induced membrane technique (IMT) is the first step of the Masquelet technique, which was described by Masquelet in 2010 and can provide essential stabilization for compound tissue repair [1, 2]. Traditionally, spongy bone grafts have generally been recommended in the second-stage process of the Masquelet technique [3, 4]. Because of the reliability, simplicity, and replicability of the IMT in the treatment of segmental bone defects in long bones, it has become a common treatment option worldwide in the last decade [1, 5]. Nevertheless, there is a paucity of literature regarding its use to treat defects of the metacarpal and phalanx.

Although segmental bone defects are not more than 5 cm, which are acknowledged as critical defects in long bones [6], these defect are caused by traumatic injury, infection or tumor debridement in the hand and are also common; the length of the affected bone can even be more than 2-2.5 times the diameter [7, 8]. It is also a challenge for hand surgeons to address these defects in emergency surgery or in the context of posttraumatic osteomyelitis; in particular, segmental bone defects are usually concomitant compound injuries of the hand that need a simple, rapid, and stable bone structure, and the outcome is much more attractive if the treatment has the potential to prevent the risk of infection or resist infection [9, 10]. Unquestionably, bone stabilization plays a fundamental role in the repair of involved tendons, nerves, blood vessels, and microvascular flaps and in rehabilitation. At the second stage, the granulated cancellous bone compactly fills the space of the defect; however, the disadvantage associated with this approach is the sacrifice of inherent bone graft stability.

In this study, we present the application of this technique in the hand with a prefabricated, cylindrical, structural iliac bone-like graft, not spongy bone material, for the loss of a phalanx or metacarpal. The feasibility and the clinical outcomes of treatment were evaluated by radiography during follow-up.

## Materials and methods

The study complies with the ethical principles of the World Medical Association Declaration of Helsinki for Medical Research Involving Human Subjects. Informed consent was obtained from all patients preoperatively. After we obtained institutional review board approval, we retrospectively examined sixteen consecutive patients who sustained phalangeal or metacarpal segmental bone defects and were treated by the IMT and autologous strip bone grafting at our center between June 2020 and June 2021 (Table 1). Thirteen men and three women, with a mean age of 40.9 years (27–60 years), presented with hand injuries, including bone loss and tendon, nerve, and blood vessel damage, and even microvascular flaps. The mechanisms of injury were as follows: crush injury (6 cases), roller machine injury (3 cases), saw injury (6 cases), and bar cutting machine injury (1 case). The metacarpal was involved in 9 cases, the phalanx in 7 cases, the left hand in 6 cases, and the right hand in 10 cases. All of the fractures were open fractures; there were eleven acute bone defects and four cases of posttraumatic osteomyelitis after open fracture. The segmental bone defect ranged from 1 ⋅ 1 ⋅ 1 cm to 5 ⋅ 1.2 ⋅ 1 cm, and the structural bone graft ranged from 1 ⋅ 1 ⋅ 1 cm to 5 ⋅ 1.2 ⋅ 1 cm.

**Table 1:** Patient demographic and surgical details

Patient	Sex	Age (years)	Side	Injury	Involved bone	Defect size (cm ⋅ cm)	Bone graft (cm ⋅ cm)
1	M	46	R	Crush injuries/osteomyelitis	Right fourth metacarpal	2⋅1⋅0.8	2⋅1⋅1
2	M	32	R	Roller machine injuries	Distal phalanx of the right thumb	2.5⋅1.1⋅0.8	2.5⋅1.1⋅0.8
3	M	30	R	Saw injuries	Middle phalanx of the right middle finger	2.7⋅1.2⋅1	2.7⋅1.2⋅1
4	M	56	R	Roller machine injuries	Proximal phalanx of the right index finger	2⋅1.2⋅1	2⋅1.2⋅1
5	M	33	R	Crush injuries/osteomyelitis	Proximal phalanx of the right little finger	1.2⋅1⋅1	1.2⋅1⋅1
6	F	33	L	Crush injuries	Left first metacarpal	1⋅1⋅1	1⋅1⋅1
7	M	30	R	Roller machine injuries/osteomyelitis	Middle phalanx of the right ring and little finger	1.8⋅1.3⋅1 (ring), 2.0⋅1.3⋅1 (little)	1.8⋅1.3⋅1 (ring), 2.0⋅1.3⋅1 (little)
8	M	56	R	Saw injuries	Right second and third metacarpals	4⋅1.4⋅1 (second), 5⋅1.2⋅1 (third)	4⋅1.4⋅1 (second), 5⋅1.2⋅1 (third)
9	M	35	R	Saw injuries	Right second and third metacarpal	1.5⋅1⋅1(second), 2.5⋅1⋅1(third)	1.5⋅1⋅1(second), 2.5⋅1⋅1(third)
10	M	54	L	Crush injuries	Left first metacarpal	1.5⋅1.4⋅1	1.5⋅1.4⋅1
11	M	27	R	Crush injuries	Proximal phalanx of the right middle finger	4⋅1.2⋅1	4⋅1.2⋅1
12	M	53	L	Crush injuries	Proximal phalanx of the left thumb	2⋅1.5⋅1	2⋅1.5⋅1
13	M	38	L	Saw injuries	Middle phalanx of the left index finger	1.5⋅1⋅1	1.5⋅1⋅1
14	F	29	L	Bar cutting machine/osteomyelitis	Distal and middle phalanges of the left ring finger	2⋅1⋅1	2⋅1⋅1
15	M	60	L	Saw injuries	Left first metacarpal	2.5⋅1.5⋅1	2.5⋅1.5⋅1
16	M	42	R	Crush injuries	Right second metacarpal	3.4⋅1.2⋅1	3.4⋅1.2⋅1
Mean	-	40.9	-	-	-	-	-

All patients in the study were required to meet the following criteria: (1) acute open fracture with tendon, nerve, vessel, or tissue defects requiring reconstruction, with the need for bone stability and preventive measures against infection; and (2) chronic osteomyelitis after open fracture with a segmental bone defect requiring reconstruction and the need for bone stability and anti-infective therapy.

### Surgical procedure

The operation was performed under general anesthesia with tourniquet control.

In the first step, absolute and extensive debridement of the injured hand or infected site was performed. Free bone fragments were removed from the site, and rigid fixation of the remaining segments was performed for the injured bone. Then, tendon, nerve, vessel and tissue defects were repaired in sequence. Joint fusion was performed in the case of articular cartilage defects or infection. Then, the defect was measured, and cement (Heraeus, Hanau, Germany) was fabricated into a cylindrical shape similar to the shape of the metacarpal or phalanx. According to the bacteria found and the pathological results, 0.55 g of gentamicin or 1.5 g of vancomycin per 40 g of bone cement was added. Kirschner wires were crossed in advance of the cylindrical cement before the bone cement set. The bone ends were wrapped for several millimeters with lamellar cement. During the procedure of cement setting, high temperatures were prevented with normal saline irrigation; then, solid fixation of the cement in the space of the bone defect was performed.

**Fig. 1:**
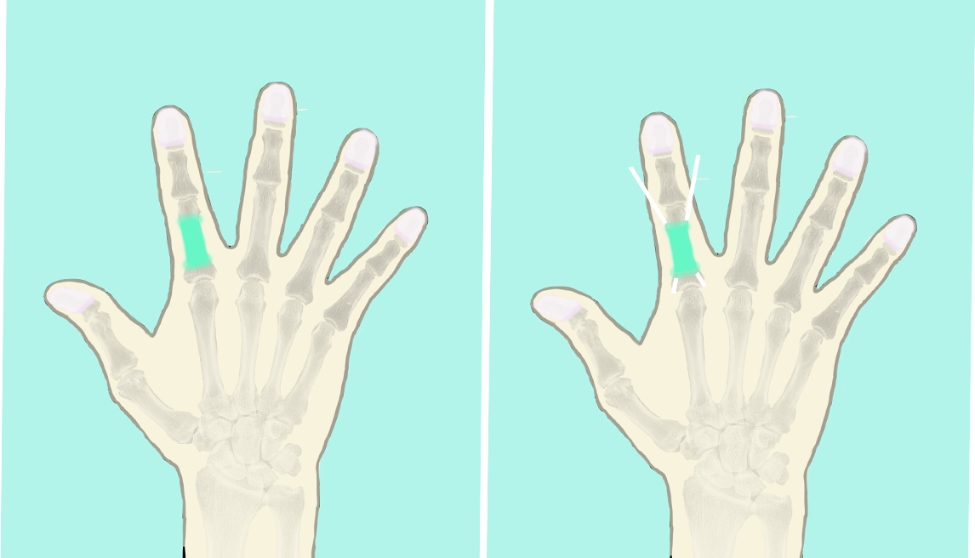
**A**, Antibiotic bone cement was fabricated into a shape similar to that of the phalanx and was utilized to fill the segmental defect, and the bone ends were wrapped for approximately one centimeter beyond the acute traumatic bone with lamellar cement. **B**, Kirschner wires were crossed to fix the bone cement

**Fig. 2:**
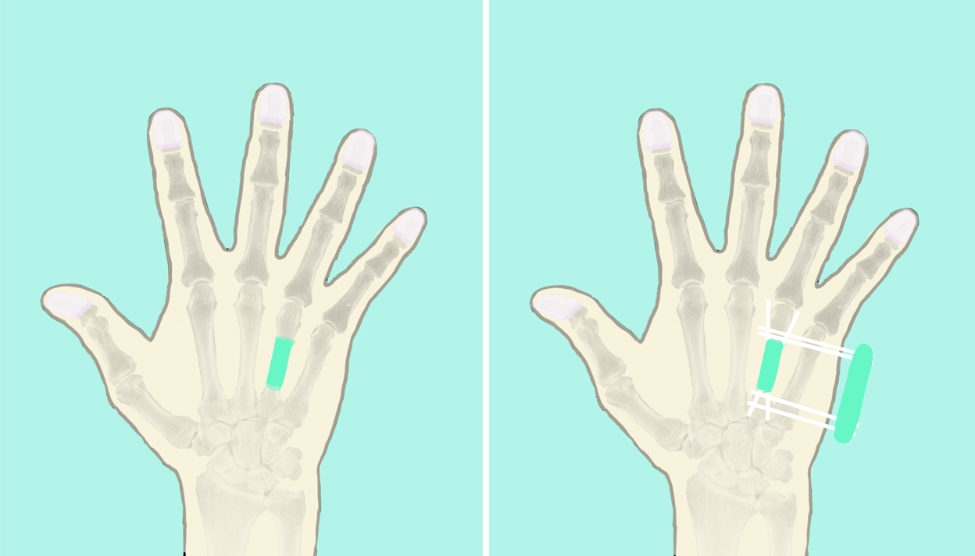
**A**, Antibiotic bone cement was fabricated into a shape similar to that of the metacarpal and was utilized to fill the segmental defect, and the bone ends were wrapped for approximately one centimeter. **B**, Several transverse and parallel Kirschner wires were placed for solid fixation with the adjacent metacarpal

For patients with osteomyelitis, unhealthy bone affected by infection was completely debrided from the healthy bone until bleeding was observed. Antibiotic bone cement with a cylindrical shape similar to that of the metacarpal or phalanx was utilized to fill the segmental defect, and the bone ends were wrapped for approximately one centimeter beyond the acute traumatic bone defect with lamellar cement (Fig. 1). Solid fixation was then achieved using several transverse and parallel Kirschner wires fixed to the adjacent metacarpal (Fig. 2).

In the second step, at 8 weeks postoperatively, there were no signs of infection, and no swelling was exhibited in the local soft tissues. The induced membrane was exposed along the original incision and then incised along the long axis, maintaining the surrounding membrane, and the antibiotic spacer was carefully removed. Nonvascular structural bone was harvested from the iliac crest, including cancellous and cortical bone. Then, the bone was carved into a cylindrical shape similar to the shape of the metacarpal or phalanx, retaining part of the cortical bone as the dorsal face at the site. Crossed or transverse and parallel Kirschner wires were used for rapid and rigid fixation of the bone end. The bone was completely wrapped by the induced membrane, and the induced membrane and the wound were closed.

### Postoperative treatment

Laboratory tests, including routine blood, erythrocyte sedimentation rate (ESR) and C-reactive protein tests, were performed regularly to evaluate indicators of inflammation or infection. Plaster was used to prevent motion for four weeks, and guided functional exercise of the healthy finger was performed. Follow-up X-ray examinations were regularly performed 4 weeks after surgery and every 2 weeks thereafter until fracture union was achieved with bony callus bridge formation evident on X-ray. The Kirschner wires were removed, and functional rehabilitation exercise of the affected finger was carried out.

### Data collection

The bone union time was recorded. The pain of the bone graft donor site was self-assessed by the patients using the visual analog scale (VAS), with a score ranging from 0 (no pain) to 10 (intense pain). The outcomes were divided into three levels, as follows: good, < 4; fair, 7 − 4, poor, 10 − 8). The total active motion (TAM) scoring system of the American Society for Surgery of the Hand was used to assess the injured finger. The parameters were compared with those of the opposite side, as follows (100%, normal, excellent; 75-99%, normal, good; 50-74%, normal, fair; <50% normal, poor).

## Results

The average follow-up was 24 weeks (range, 12–40 weeks). All bone grafts showed union after an average of 8.6 weeks (range, 8–12 weeks) on radiography, and bone resorption was noted on X-ray examinations. All incisions at donor and recipient sites demonstrated primary healing without subsequent infection complications. One patient complained of pain at the bone donor site, and no patients complained of pain at the recipient site. There were no cases of secondary infection, bone resorption, or nonunion at the bone graft site. The mean VAS score of the donor site was 1.8 (range, 0–5), with 13 good scores and 3 fair scores. The mean TAM of the fingers was 179.9° (range, 90°-235°), including 4 excellent, 8 good, and 4 fair results (Table 2).

**Table 2:** Outcomes of the involved hand at the final follow-up

Patient	Bone union time (weeks)	Follow-up time (weeks)	VAS score	TAM	Compared with the opposite side (%)
1	8	24	2	235	100
2	8	22	0	90	50
3	8	33	0	135	57
4	8	28	3	210	89
5	8	32	0	200	85
6	8	24	2	180	100
7	8	17	4	200	85
8	12	20	5	185	78
9	8	15	3	180	100
10	12	18	0	210	89
11	8	24	1	158	67
12	8	16	0	130	72
13	8	28	0	186	79
14	10	33	2	190	80
15	10	12	4	180	100
16	8	40	3	210	89
Mean	8.6	24.1	1.8-	179.9	-

### Case reports

#### Case 1

**Fig. 3:**
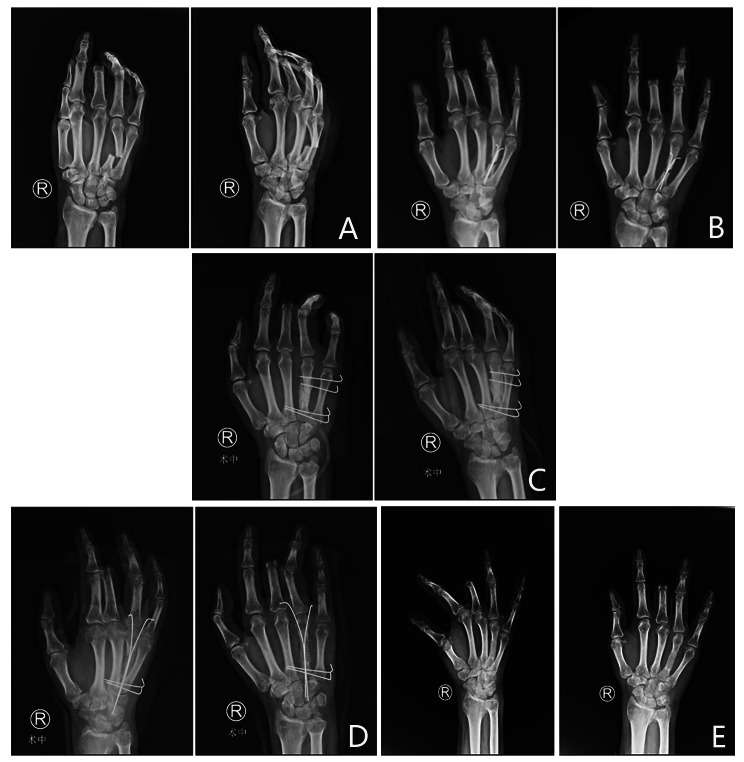
The right hand of a 46-year male patient was crushed by a machine, resulting in open fractures with nerve, vessel, and soft tissue defects. **A**, Preoperative X-ray. **B**, X-ray at four months postoperatively, indicating posttraumatic osteomyelitis in his fourth metacarpal. **C**, After the sinus was removed, the bone defect was filled with antibiotic PMMA cement, and the cement was fabricated into a cylindrical shape similar to the shape of the metacarpal. Four transverse and parallel Kirschner wires were placed for fixation with the adjacent metacarpal. **D**, At 8 weeks postoperatively, the cement spacer was removed, and the bone graft was carved into a 2 cm⋅ 1 cm ⋅ 1 cm cylindrical shape similar to the shape of the metacarpal. Two crossed Kirschner wires were used to fix the fracture. **E**, At eight weeks postoperatively, bone graft union was observed without additional infection

The right hand of a 46-year male patient was crushed by a machine, causing soft tissue and tendon defects and fracture of the fourth metacarpal. During his first operation, he underwent acute tendon suturing, bone fixation with K-wires and soft tissue suturing. Two months later, posttraumatic osteomyelitis was diagnosed in his fourth metacarpal through radiography and CT examination (Fig. 3). After the sinus, necrotic tissue and dead bone were removed, the defect was filled with antibiotic PMMA cement, and the cement was fabricated into a cylindrical shape similar to the shape of the metacarpal. Four Kirschner wires were placed transverse and parallel for fixation to the adjacent metacarpal. At 8 weeks, the cement spacer was removed, and the bone graft was carved into a 2 cm⋅ 1 cm ⋅ 1 cm cylindrical shape similar to the shape of the metacarpal. Two crossed Kirschner wires were used to fix the fracture. At eight weeks postoperatively, bone graft union was present without additional infection. At the 24-week follow-up, the TAM of the injured finger was 200°, and the VAS score of the bone graft donor site was 2.

#### Case 4

**Fig. 4:**
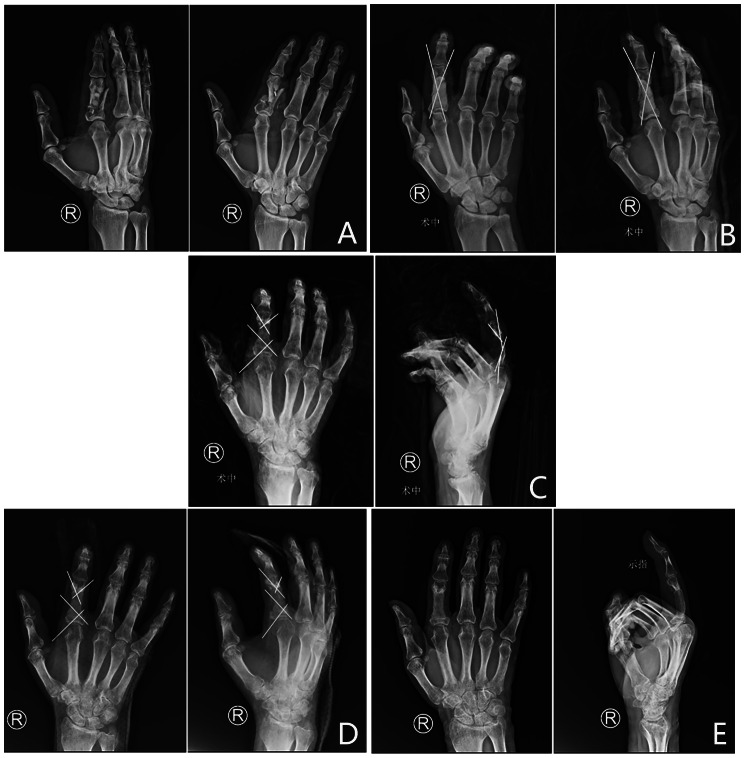
The right index finger of a 56-year male patient was impaired by a roller machine, resulting in open fracture of the proximal phalanx with tendon, nerve, vessel, and soft tissue defects. **A**, Preoperative X-ray. **B**, After debridement, the proximal phalanx presented a segment defect, and cement shaped like the phalanx was used to fill the space. **C**, At 8 weeks postoperatively, the cement spacer was removed, and the bone graft was carved into a 2.0 cm ⋅ 1.2 cm ⋅ 1.0 cm cylindrical shape similar to the shape of the metacarpal. Two crossed Kirschner wires were used to fix the fracture. **D**, At eight weeks postoperatively, callus formation was observed. **E**, At 8 weeks postoperatively, bone graft union was observed without additional infection

The right index finger of a 56-year male patient was impaired by a roller machine, causing defects of the proximal phalangeal segment, soft tissue, and tendon, as well as proper digital artery and nerve injury. During his first operation, he underwent acute tendon suturing, soft tissue suturing, proper digital artery anastomosis and nerve repair, and the bone defect was filled with cement shaped like the phalanx (Fig. 4). At 8 weeks, the cement spacer was removed, and the bone graft was carved into a 2.0 cm ⋅ 1.2 cm ⋅ 1.0 cm cylindrical similar to the shape of the phalangeal. Two crossed Kirschner wires were used to fix the fracture. At eight weeks postoperatively, bone graft union was present without additional infection. At the 28-week postoperative follow-up, the TAM of the injured finger was 210°, and the VAS score of the bone graft donor site was 3.

#### Case 14

**Fig. 5:**
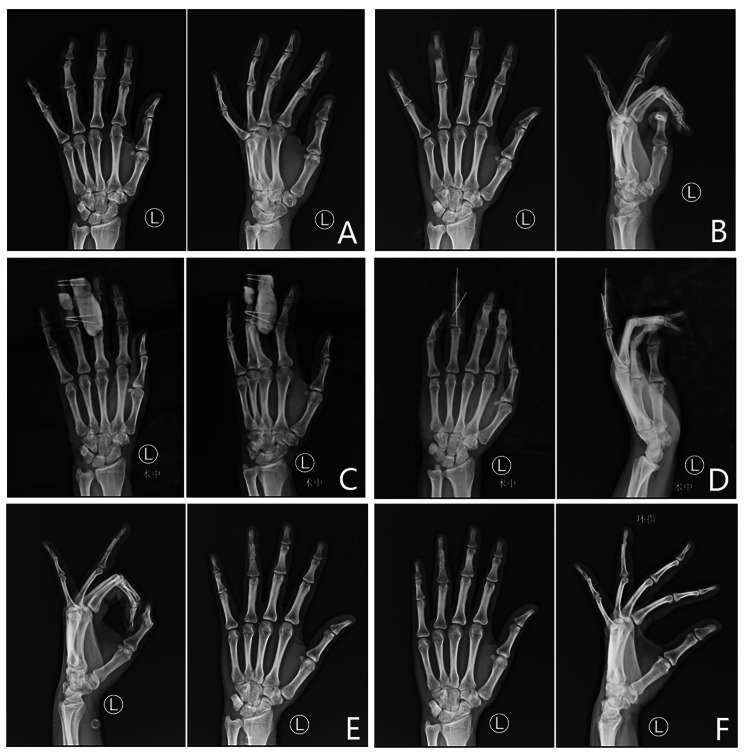
The left ring finger of a 29-year female patient was injured by a bar cutting machine, causing open injury of the distal interphalangeal joint and tendon, nerve, vessel, and soft tissue defects. **A**, Preoperative X-ray. **B**, Subsequent posttraumatic osteomyelitis. **C**, After the sinus and dead bone were removed, the defect was filled with antibiotic PMMA cement fabricated into a cylindrical shape similar to the shape of the phalanx. External fixation was achieved with four transverse and parallel Kirschner wires and additional cement to connect the Kirschner wires. **D**, At 8 weeks postoperatively, the cement spacer was removed, and the bone graft was carved into a 2.0 cm ⋅ 1.0 cm ⋅ 1.0 cm cylindrical shape similar to the shape of the phalanx. Two Kirschner wires were used to fix the fracture. Distal interphalangeal joint fusion was performed. **E**, At four weeks postoperatively, callus formation was observed. **F**, At 10 weeks postoperatively, bone graft union was observed without additional infection

The left ring finger of a 29-year female patient was injured by a bar cutting machine, causing open damage to the distal interphalangeal joint, soft tissue and tendon defects, and fracture of the middle phalanx. At her first operation, she underwent acute tendon suturing, bone fixation with Kirschner wires and free arterialized flap transfer for soft tissue coverage. Five weeks later, posttraumatic osteomyelitis was diagnosed in her distal and middle phalanges through radiography (Fig. 5). After the sinus and dead bone were removed, the defect was filled with antibiotic PMMA cement. The cement was fabricated into a cylindrical shape similar to the shape of the phalanx. External fixation was achieved with four transverse and parallel Kirschner wires and additional cement to connect the Kirschner wires. At 8 weeks postoperatively, the cement spacer was removed, and the bone graft was carved into a 2.0 cm ⋅ 1.0 cm ⋅ 1.0 cm cylindrical shape similar to the shape of the phalanx. Two Kirschner wires were used to fix the fracture. Distal interphalangeal joint fusion was performed. At 10 weeks postoperatively, bone graft union was observed without additional infection. At the 33-month follow-up, the TAM of the injured finger was 190°, and the VAS score of the bone graft donor site was 1.

## Discussion

Our outcomes demonstrate that IMT with a nonvascular structural bone including cancellous and cortical bone that was carved into a cylindrical shape similar to the shape of the metacarpal or phalanx, instead of spongy bone, to fill segmental bone defects of the hand due to acute open fracture or posttraumatic osteomyelitis could provide immediate bone stabilization and structural support in orthopedic bone defects for repairing the involved tendons, nerves, blood vessels, and microvascular flaps, facilitating subsequent rehabilitation, reducing scar tissue formation, and preventing soft tissue contracture in the second stage of the bone grafting procedure [11]. 100% bone union was achieved in the patients at an average of 8.6 weeks postoperatively. Early rehabilitation exercises could be performed, and good functional outcomes could be acquired.

In the traditional Masquelet technique, spongy bone is recommended due to its good bone osteoconductivity, osteoinductivity, and osteogenic activity [3], and the cancellous bone is granulated to compactly fill the space. However, the disadvantage associated with this approach is the sacrifice of inherent bone graft stability. Cortical bone grafts supply minimal osteoinductive and osteogenic properties but provide immediate mechanical stability [12]. In our study, the structural cylindrical bone graft, which contained cancellous bone on three faces and cortical bone on one face, was carved into a shape similar to the shape of the metacarpal or phalanx and was utilized in the second stage. It not only exhibited strong osteoinductive and osteogenic properties and was mostly osteoconductive but also provided immediate mechanical stability and adequate mechanical strength to allow for early finger movement [13]. With the benefits of the rich blood supply and osteogenic environment of the IM, no cases of bone absorption or bone nonunion were observed within the follow-up period.

Additionally, with the aid of the IM, which offers a regenerative microenvironment including abundant growth factors, such as vascular endothelial growth factor (VEGF), transforming growth factor-β1 (TGF-β1), interleukin 6 (IL-6), interleukin 8 (IL-8), and bone morphogenetic protein-2 (BMP-2), the vascular bed acts as a protective capsule-like layer [14], which is an ideal environment for bone union and regeneration. The low osteoinductivity was compensated for by the IM, and the bone healing time and the bone union rate, as demonstrated on follow-up radiography, were ideal. We make a guess at the style of the bone union, maybe it is not a common creeping substitution, and is union directly.

Antibiotic-loaded bone cement provides a local high dose of antibiotics in the treatment of infection and prevention of infection in the case of an open fracture [10, 15]. By reconstructing the segmental defect with a cylindrical cement graft shaped similar to the metacarpal or phalanx, a space similar to the void of the original bone can be created. Additionally, the bone ends were wrapped for approximately one centimeter beyond the length of the cylindrical graft with lamellar cement to create an IM with adequate space for the bone graft and achieve a capsule-like layer to enclose all of the autografted bone. Hence, maintaining the intact IM is also vital, and very careful removal of the cement spacer is needed. The volume of the bone graft is another vital factor, and we advocate fabricating the cement spacer with a volume slightly larger than the defect of the metacarpal or phalanx to guarantee sufficient space for the elaborately carved bone graft in the actual defect of the metacarpal or phalanx. Providing an independent and relatively closed microenvironment for bone healing and preventing the growth and migration of soft tissues were prerequisites.

Although nonvascular bone grafting is a common technique for the reconstruction of defects smaller than 4–6 cm that would allow healing [7, 16], the IMT provides an attractive microenvironment with unrivaled bioactivity of various and adequate proteins for protecting and promoting osteogenesis of the IM; therefore, the IMT provides much better conditions for achieving bone union than traditional iliac crest bone grafting [17]. All patients in the series achieved bone union after a mean of 8.6 weeks, including patients with emergency traumatic bone defects and posttraumatic osteomyelitis.

Free vascularized bone grafting is usually used for the treatment of bone defects [18–22]; however, the necessary fundamental technique of vascular anastomosis is a limitation. The reverse metacarpal bone flap has been reported, with bone healing achieved in six weeks [23]; nevertheless, the procedure for bone flap dissection is time consuming, and strong anatomical skills are required. Although the patients underwent two surgeries in this therapeutic process, there were no cases of subsequent correlative infection complications in any patient, even patients with osteomyelitis. The IMT presented reliable efficacy in preventing and controlling infection [23].

Complications at the iliac bone graft harvest site should not be ignored [24]. The present study was a respective study, and the small number of patients is a limitation. Additionally, we only concentrated on the fracture healing status, VAS score, TAM, infection complications, lack of shortening, grip strength and other evaluation indicators; multiple additional dimensions need to be evaluated over longer-term follow-up in the future. Multicenter studies and comparative studies should also be performed to obtain more data in the future.

## Conclusions

In conclusion, the treatment of segmental bone defects of the metacarpal or phalanx due to acute open fracture or osteomyelitis with the IMT and a structural cylindrical bone graft was demonstrated by follow-up radiography results. The bone graft provided much more stability and structural support in the orthopedic bone defects, and the evaluated outcomes showed that the bone healing time and the bone union rate were ideal.

## Data Availability

The datasets supporting the conclusions of this article are included within the article. Raw data can be requested from the corresponding author.

## Abbreviations

VAS: Visual analog scale
TAM: Total active motion
VEGF: Vascular endothelial growth factor
IL-6: Interleukin 6
IL-8: Interleukin 8
TGF-β1: Transforming growth factor-β1
BMP-2: Bone morphogenetic protein-2
